# On the molecular and cellular effects of omeprazole to further support its effectiveness as an antigiardial drug

**DOI:** 10.1038/s41598-019-45529-w

**Published:** 2019-06-20

**Authors:** Gabriel López-Velázquez, Cynthia Fernández-Lainez, José Ignacio de la Mora-de la Mora, Daniela Caudillo de la Portilla, Rafael Reynoso-Robles, Angélica González-Maciel, Cecilia Ridaura, Itzhel García-Torres, Pedro Gutiérrez-Castrellón, Alfonso Olivos-García, Luis Antonio Flores-López, Sergio Enríquez-Flores

**Affiliations:** 1 Grupo de Investigación en Biomoléculas y Salud Infantil, Laboratorio de EIMyT, Instituto Nacional de Pediatría, Ciudad de México, 04530 Mexico; 2 Laboratorio de Errores Innatos del Metabolismo y Tamiz, Instituto Nacional de Pediatría, Ciudad de México, 04530 Mexico; 3 Laboratorio de Morfología Celular y Tisular, Instituto Nacional de Pediatría, Ciudad de México, 04530 Mexico; 4 Departamento de Patología, Instituto Nacional de Pediatría, Ciudad de México, 04530 Mexico; 5 Hospital General Dr. Manuel Gea González, Ciudad de México, 14080 Mexico; 6 Unidad de Investigación en Medicina Experimental, Facultad de Medicina, Universidad Nacional Autónoma de México y Hospital General, Ciudad de México, 04510 Mexico; 7 CONACYT-Instituto Nacional de Pediatría, Secretaría de Salud, Ciudad de México, 04530 Mexico

**Keywords:** Enzymes, Infectious diseases

## Abstract

Research on *Giardia lamblia* has accumulated large information about its molecular cell biology and infection biology. However, giardiasis is still one of the commonest parasitic diarrheal diseases affecting humans. Additionally, an alarming increase in cases refractory to conventional treatment has been reported in low prevalence settings. Consequently, efforts directed toward supporting the efficient use of alternative drugs, and the study of their molecular targets appears promising. Repurposing of proton pump inhibitors is effective *in vitro* against the parasite and the toxic activity is associated with the inhibition of the *G. lamblia* triosephosphate isomerase (*Gl*TIM) via the formation of covalent adducts with cysteine residue at position 222. Herein, we evaluate the effectiveness of omeprazole *in vitro* and *in situ* on *Gl*TIM mutants lacking the most superficial cysteines. We studied the influence on the glycolysis of *Giardia* trophozoites treated with omeprazole and characterized, for the first time, the morphological effect caused by this drug on the parasite. Our results support the effectiveness of omeprazole against *Gl*TIM despite of the possibility to mutate the druggable amino acid targets as an adaptive response. Also, we further characterized the effect of omeprazole on trophozoites and discuss the possible mechanism involved in its antigiardial effect.

## Introduction

Giardiasis is caused by *Giardia lamblia*, which is one of the most common infectious protozoans on the globe and is responsible for diarrheal disease and chronic postinfectious illnesses such as irritable bowel syndrome^1^. The clinical impact of giardiasis seems to be stronger in the first 3 years of life and in undernourished or immunodeficient individuals^2,3^. This parasitic disease continues to be the major cause of nonviral or bacterial diarrhea in humans and other vertebrates^4–6^. Its prokaryotic‐like anaerobic metabolism renders it selectively sensitive to certain bacterial drugs, especially nitroimidazoles, which are activated to form toxic radicals. Nevertheless, the drugs used to treat giardiasis have associated side effects, and drug resistance has been demonstrated or induced *in vitro*^7,8^.

The ability of *Giardia* cysts to persist in the environment, including in the presence of disinfectants^9,10^, and the existence of lethal strains with the potential to infect humans^11,12^ is strikingly important in the context of bioterrorism. Altogether, the features of giardiasis highlight the importance of this disease as a public health problem and have led to a search for novel experimental strategies and evaluation of alternative treatment regimens.

*G. lamblia* is characterized by its lack of mitochondria and cytochrome-mediated oxidative phosphorylation. The parasite relies on both glycolysis and arginine dihydrolase pathways for energy generation, even in the presence of oxygen^13^. *Giardia* optimizes glycolysis by using pyrophosphate (PPi) instead of adenosine monophosphate (AMP) as a phosphate donor, which allows generation of five ATP molecules rather than the two yielded by the common Embden-Meyerhof-Parnas pathway^14^. However, some authors claim that arginine is a major potential energy source during the initial stages of giardial growth^15–17^.

Triosephosphate isomerase (EC 5.3.1.1) is a key enzyme in glucose and glycogen metabolism^18^, and we previously demonstrated the mechanism by which this enzyme in *G. lamblia* (*Gl*TIM) is inactivated by chemical modification with thiol-reactive compounds and proposed it as a target for drug design^19,20^. Therefore, we hypothesize that when *Gl*TIM is inactivated an energetic imbalance is provoked, which might be harmful for the parasite due to the effect on glycolysis and accumulation of toxic metabolites, such as methylglyoxal.

Previously, we demonstrated the species-specific effect of omeprazole as a *Gl*TIM inhibitor and its concomitant cytotoxic effect on trophozoites^21^. Each *Gl*TIM monomer (the functional unit of this enzyme can be a dimer or tetramer)^22^ possesses 5 cysteine (Cys) residues at positions 14, 127, 202, 222, and 228, with Cys 202 and 228 being the most exposed to the milieu^19^. In addition, we demonstrated that the Cys at position 222 of *Gl*TIM is the main target in the inhibition of the enzyme by omeprazole (OMP)^21^ and other commercial proton pump inhibitors (PPIs)^23^. On one hand, OMP interacts with 3 Cys in the WT enzyme and in the single C14A and C127A mutants; on the other hand, OMP interacts with 2 Cys in the single mutants C202A, C222A, and C228A^24^. Therefore, the participation of Cys 222 in *Gl*TIM inactivation is well known, but the roles of the remaining 4 Cys in the enzyme are still unknown.

Antimicrobial drugs have demonstrated remarkable effectiveness in the control of parasite infections. However, it is evident that pathogens are unlikely to surrender unconditionally because they rapidly become resistant to many of the effective first-line drugs. To further expand knowledge of the molecular mechanisms involved in the effect of OMP on *Gl*TIM and examine its effect at the cellular level, we aimed to establish the susceptibility of *Gl*TIM to OMP *in vitro* and *in situ* if the parasite mutated the Cys residue with the highest accessible surface area (ASA) (*i.e*., superficial Cys more easily targeted by OMP) in a natural process to avoid inhibition of its enzyme. Additionally, we analyzed the effect of this drug at the ultrastructural level in *G. lamblia* trophozoites.

Here, we demonstrate that absence of the superficial Cys in *Gl*TIM slows the inactivation process exerted by OMP but is not sufficient to avoid it. Using bacteria transformed by wild-type and mutant genes coding for the abovementioned *Gl*TIMs as alternative cellular models, we were able to test the effect of OMP on these enzymes in the cell. We propose that OMP truncates glycolysis in *G. lamblia* and induces strong structural damage to trophozoites prior to cell death. Altogether, our results support our proposal that potential mutation of the superficial Cys in the parasite TIM would be insufficient to avoid the deleterious effects of OMP on *Gl*TIM. Although OMP potentially truncates glycolysis in *Giardia*, the cytotoxic effect appears to be driven by the production of methylglyoxal and accumulation of advance glycation end products (AGEs) rather than by an energetic imbalance related to *Gl*TIM inhibition. However, many other unidentified proteins are also targeted by omeprazole, and their contribution to the cytotoxic effect of OMP should be evaluated in future studies.

## Results

### Recombinant *Gl*TIM mutants were efficiently produced and exhibited no major structural differences but their thermal stability and kinetics were altered

Wild-type (WT) *Gl*TIM and double (Dmut) and triple (Tmut) mutants were efficiently purified to homogeneity; the His-tag and tobacco etch virus (TEV) protease cleavage systems yielded approximately 140 (range 70–250), 300 (range 250–380), and 108 (range 55–190) mg of pure recombinant protein per liter of cell culture, respectively. All the recombinant proteins showed Michaelis-Menten kinetics with the constants shown in Table 1. In comparison with *Gl*TIM WT, the affinity of DMut was twice increased, whereas that of Tmut was decreased by almost half. The turnover rate (*k*_cat_) of Dmut was lower but similar to that of WT, whereas Tmut was about half that of WT. Additionally, the overall kinetics of Tmut resembled those of *Gl*TIM derivatized with the thiol reactive reagent methyl methanethiosulfonate (MMTS) as previously demonstrated^19^.

**Table 1:** Kinetic and stability constants for *Gl*TIM WT and double (Dmut) and triple (Tmut) mutants compared with *Gl*TIM derivatized with methyl methanethiosulfonate (MMTS) and *Gl*TIM tetramer. ND, not determined.

Enzyme	*K*_m_ (mM)	*k*_cat_ (10^5^ min^−1^)	*k*_cat_/*K*_m_ (mM^−1^min^−1^)	Tm_app_ (°C)	Reference
*Gl*TIM WT	0.78 ± 0.06	4.6 ± 0.16	5.9 × 10^5^	58	This work
DMut C222A-C228A	0.38 ± 0.03	3.78 ± 0.12	9.9 × 10^5^	54.5	This work
Tmut C202A-C222A-C228A	1.2 ± 0.09	2.35 ± 0.14	1.15 × 10^5^	55.9	This work
*Gl*TIM WT + MMTS	0.97 ± 0.1	1.9 ± 0.08	1.96 × 10^5^	54	Enríquez-Flores, *et al*.^19^
*Gl*TIM tetramer	0.87 ± 0.07	1.47 ± 0.04	1.69 × 10^5^	ND	López-Velázquez, *et al*.^22^

The data represent the mean of four independent experiments.

**Figure 1:**
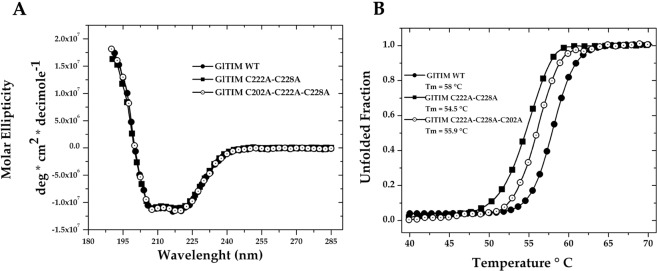
Spectroscopic properties of *Gl*TIM WT, Dmut (C222A-C228A), and Tmut (C202A-C222A-C228A). Far-UV CD spectra (**A**) and thermal denaturation (**B**).

Characterization of both Dmut and Tmut indicated that the mutations induced minor changes in the secondary structure, indicated by the similarity of the far-UV circular dichroism (DC) spectra between these mutants and the WT enzyme (Fig. 1A). Nonetheless, the thermal stability (Tm) of the two mutants decreased, with ∆Tm values of 3.5 and 2.1 °C for Dmut and Tmut, respectively (Table 1, Fig. 1B). Although marginal, the differences in Tm are likely nonconvenient features of enzymes carrying such mutations because they promote lower protein stability as previously proposed for other *Gl*TIM mutants, which showed ∆Tm values ranging from 2.3 to 6.3 °C^24^. In summary, the absence of Cys residues at positions 222 and 228 enhances substrate affinity but destabilizes the structure. Coupled with the latter, the absence of a Cys residue at position 202 strongly affects substrate affinity and ends up making it similar to a derivatized enzyme. Therefore, it seems that a loss of superficial cysteines is a thermodynamically inappropriate event for the parasite, at least at the molecular level.

### The absence of superficial Cys slows *Gl*TIM inactivation by OMP but does not prevent it

*Gl*TIM inhibition assays using single mutants of its five Cys per monomer (Supplementary Fig. S1) demonstrated that the Cys residue at position 222 is the main target for Cys-reactive compounds, including OMP^20,21^. Only the single mutant C222A (the cysteine at position 222 is substituted with an alanine) avoids inhibition by OMP^21^ and other cysteine-reactive compounds^20^. The single mutants of the remaining four cysteines (including Cys at position 14) failed to resist inactivation by OMP. In addition, under native conditions (folded protein), OMP binds only 3 cysteine residues per monomer in wild-type G*l*TIM but reaches 100% inhibition^21^, which seemingly indicates that C14 is not targeted by OMP under such conditions.

Despite this, there is no assurance that the development of new antigiardial drugs can keep pace with the ability of this pathogen to develop resistance. Strategies employed by this parasite to overcome susceptibility to Cys-reactive compounds could include single or multiple genetic mutations, leading to substitution of the amino acid residues susceptible to therapeutic drugs.

To further analyze such a possibility, it is important to revisit the behavior of the *Gl*TIM C222A mutant. When incubated for 2 h with increasing concentrations of OMP, the C222A mutant is completely insensitive to OMP until concentrations as high as 250 μM, and its activity decays 20% in the range of 300 to 750 μM, as previously demonstrated^21^. Nevertheless, after 24 h of incubation with OMP, the enzyme activity of the C222A mutant decays ∼80% (Supplementary Fig. S2). This result indicates that, rather than being the only site responsible for *Gl*TIM inhibition by OMP, Cys 222 is only one of the Cys involved in this process and the other four remaining Cys can also be targeted by OMP.

Therefore, we examined two combinations of extreme conditions related to *Gl*TIM that the parasite could adopt to defend itself against OMP: mutation of the Cys residues most susceptible to targeting by OMP based on their ASA. According to the crystallographic structure deposited in the Protein Data Bank (PDB code 2dp3), the ASA values for *Gl*TIM Cys are 49.4% (C202 *in silico* mutated), 10.5% (C228), 0.2% (C127), 0.0% (C222), and 0.0% (C14). Therefore, we studied the most superficial ones because they would be the first to interact with OMP in solution. We chose to exclude C222 because its participation in the inhibition process of recombinant G*l*TIM was already demonstrated, but we assayed the possible reactivity of C14 and C127 in the absence of the most reactive cysteine*, i.e*., cysteine 222.

**Figure 2:**
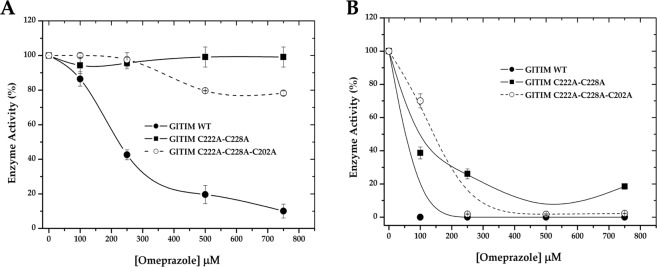
Inhibitory effect of omeprazole on recombinant giardial triosephosphate isomerase (*Gl*TIM) enzymes lacking the most superficial Cys. After incubation of the enzymes with increasing concentrations of OMP for 2 (**A**) and 24 (**B**) h at 37 °C, an aliquot was withdrawn, and the residual activity was monitored as reported in Materials and Methods. The data are the mean of at least three independent experiments. The activity registered for enzyme incubated without omeprazole was considered to represent 100% enzyme activity (2,160, 3,928, and 956 μmol min^−1^ mg^−1^ for *Gl*TIM WT, Dmut, and Tmut, respectively).

After 2 h of incubation with increasing concentrations of OMP, *Gl*TIM WT was highly sensitive to the drug; on the other hand, Dmut was insensitive to OMP, while Tmut showed behavior similar to that of the single C222A mutant (Fig. 2A). In spite of the resistance of Dmut and Tmut to OMP, after 24 h of incubation in presence of OMP, all enzymes were strongly inhibited (Fig. 2B). These results indicate that although absence of the superficial Cys slows the inhibition process it does not prevent it. Therefore, the Cys residue at either position 14 or 127 must be reached by OMP.

### Omeprazole targets and inhibits recombinant *Gl*TIM in bacterial cells

When we tried to translate this molecular model into trophozoites, we faced two challenges. (1) Endogenous *Gl*TIM in the trophozoites shows high susceptibility to OMP^21^ and would hide the effect in trophozoites transfected with the *gltim* mutant genes. (2) Genetic manipulation of *Giardia* to knock out a gene and substitute it for other is still not feasible by using the CRISPR/Cas9 system due to its two transcriptionally active diploid nuclei^25^.

**Figure 3:**
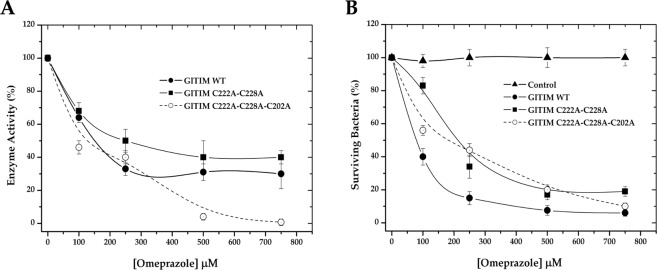
Inhibition exerted by omeprazole on *Gl*TIM in a heterologous system (**A**), and the effect of omeprazole on bacteria survival (**B**).

We chose transformed bacteria as an alternative cellular model to examine the effect of OMP on Dmut and Tmut. Since TIM from *Escherichia coli* strain BL21 has no reported sensitivity to OMP, we envisaged that bacteria transformed with the *gltim-wt, gltim-dmut*, and *gltim-tmut* genes would be a good alternative model to achieve our aim, given that glycolysis of the transformed bacteria does not depend on overexpression of heterologous TIM. To confirm that OMP reaches the heterologous enzymes in the transformed bacteria, the cultures were coincubated with increasing concentrations of OMP. After 24 h, the heterologous proteins were purified, and their enzyme activity was measured. As expected, all the recombinant proteins were reached by OMP and dose-dependently inhibited (Fig. 3A). *Gl*TIM WT and Dmut were similarly inhibited, while Tmut was more strongly affected. In an unforeseen manner, OMP not only affected the heterologous proteins but also exerted cytotoxic effects on the transformed bacteria. Survival of the nontransformed (control) and transformed bacteria with *Gl*TIM WT, Dmut, and Tmut was evaluated after 24 h of OMP exposure by reculturing in plates and counting the colony forming units (CFU). Although the *E. coli* BL21 TIM is not sensitive to OMP (Fig. 3B, control), the viability of bacteria overexpressing *Gl*TIM was strongly affected by OMP (Fig. 3B). These results suggest that the cytotoxicity exerted by OMP when it targets *Gl*TIM is not completely due to an energetic imbalance.

### Omeprazole treatment induces accumulation of PAS-positive granules and causes ultrastructural damage in *Giardia* trophozoites

**Figure 4:**
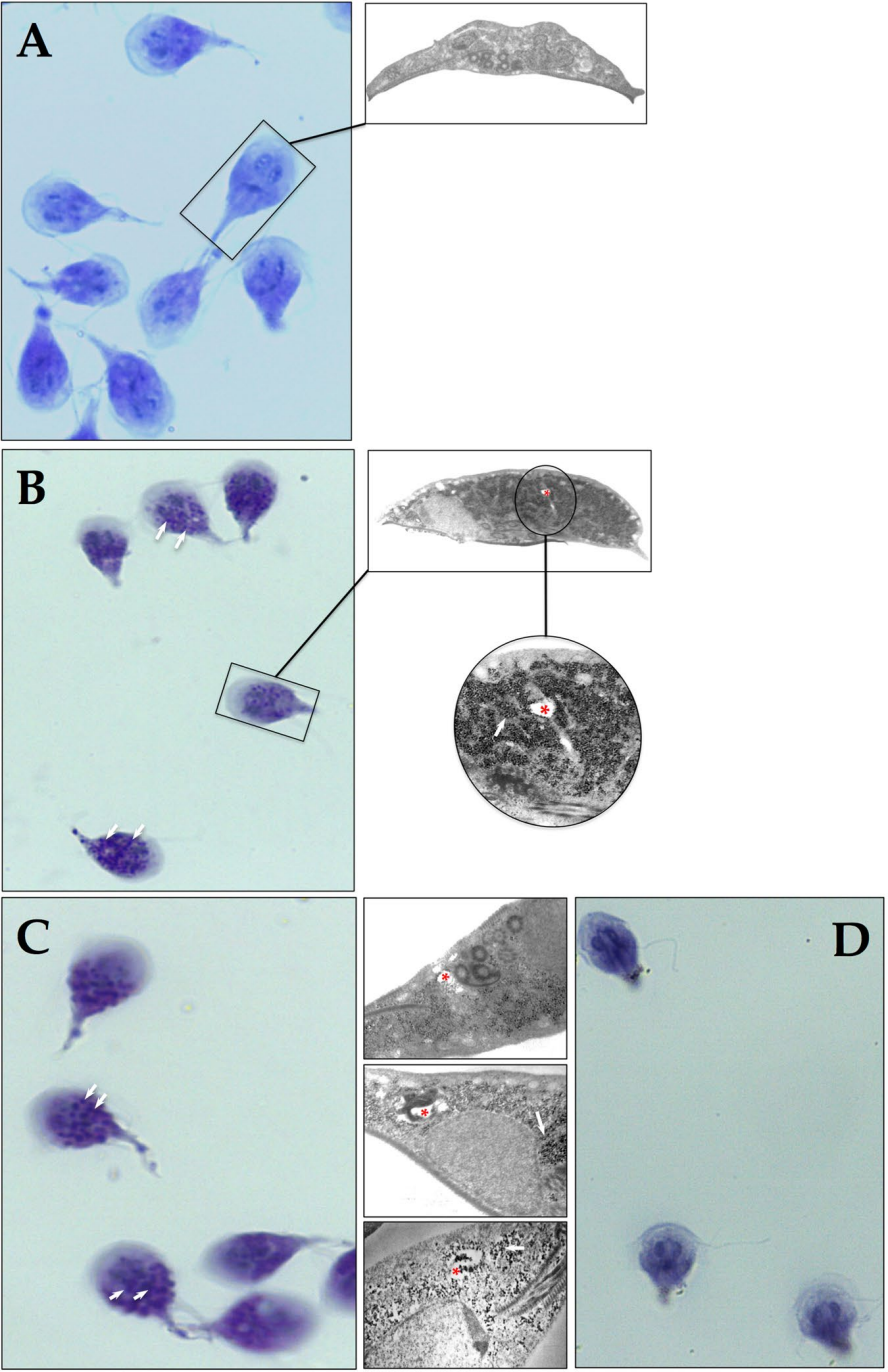
Omeprazole induces formation of PAS-positive cytoplasmic granules in *Giardia* trophozoites. *Giardia* trophozoites at log growth without OMP (**A**), exposed to 100 μM OMP for 6 h (**B**), and 500 μM OMP for 1 h (**C**) were subjected to PAS staining. Insets show the ultrastructural counterparts in each figure panel and highlight the glycogen aggregates. Trophozoites treated with OMP as in (**C**) were incubated with salivary amylase and then subjected to PAS staining (**D**). White arrows show PAS-positive granules and their ultrastructural counterparts. Red asterisks show drug-induced ultrastructural damage.

Based on the results of the present study, we reconsidered the hypothesis that glycolysis in *Giardia* is truncated through damage of *Gl*TIM caused by OMP. It is known that inhibition of glucose utilization leads to an increase in glycogen deposition, which is expected if the enzymatic pathway for generation of ATP from glucose is inhibited. Stored glycogen in *Giardia* may be detected by staining with periodic acid Schiff (PAS)^26^; thus, we performed a series of assays in which *Giardia* trophozoite cultures were incubated with 100 μM OMP for 6 h and 500 μM OMP for 1 h prior to staining with PAS (Fig. 4). It is known that cultures of *G. lamblia* trophozoites accumulate glycogen during the lag and early logarithmic phases of growth and then utilize this carbon source during their remaining logarithmic growth. As cultures enter the stationary phase of growth, they reaccumulate glycogen stores^27^. Then, trophozoites without OMP normally show some positive PAS staining (Fig. 4A). Interestingly, even after a long incubation with a low concentration (Fig. 4B) and a short incubation with a high concentration of OMP (Fig. 4C), both showed PAS-positive cytoplasmic granules.

The PAS-positive granules appear to correspond with the electron-dense granules observed in the electron microscopy photographs of trophozoites subjected to the same OMP treatments (insets in Fig. 4). Importantly, assays in which the trophozoites were treated with salivary amylase after incubation with OMP did not show PAS-positive granules after PAS staining (Fig. 4D) denoting glycogen content. Additionally, trophozoites exposed to OMP under these conditions began to show ultrastructural damage (Fig. 4, asterisk into the insets).

### Omeprazole increases methylglyoxal and Advanced Glycation End Products (AGEs) on *Giardia* trophozoites

To further analyze the G*l*TIM alteration and its contribution to the activity of omeprazole against *Giardia*, we performed a preliminary series of assays, which suggest that treatment of *Giardia* trophozoites with omeprazole increases methylglyoxal and AGEs. Since methylglyoxal is highly reactive, and the employed method detects only this metabolite in its free form, we were only able to detect differences between control cells and those treated with the highest concentration of OMP. However, as methylglyoxal rapidly links to proteins, which leads to AGEs generation, we were able to detect dose-dependent increase in these products in treated *Giardia* trophozoites (Supplementary Fig. S3).

### The IC50 value at which omeprazole induces ultrastructural damage in trophozoites is comparable with those of metronidazole and nitazoxanide

**Figure 5:**
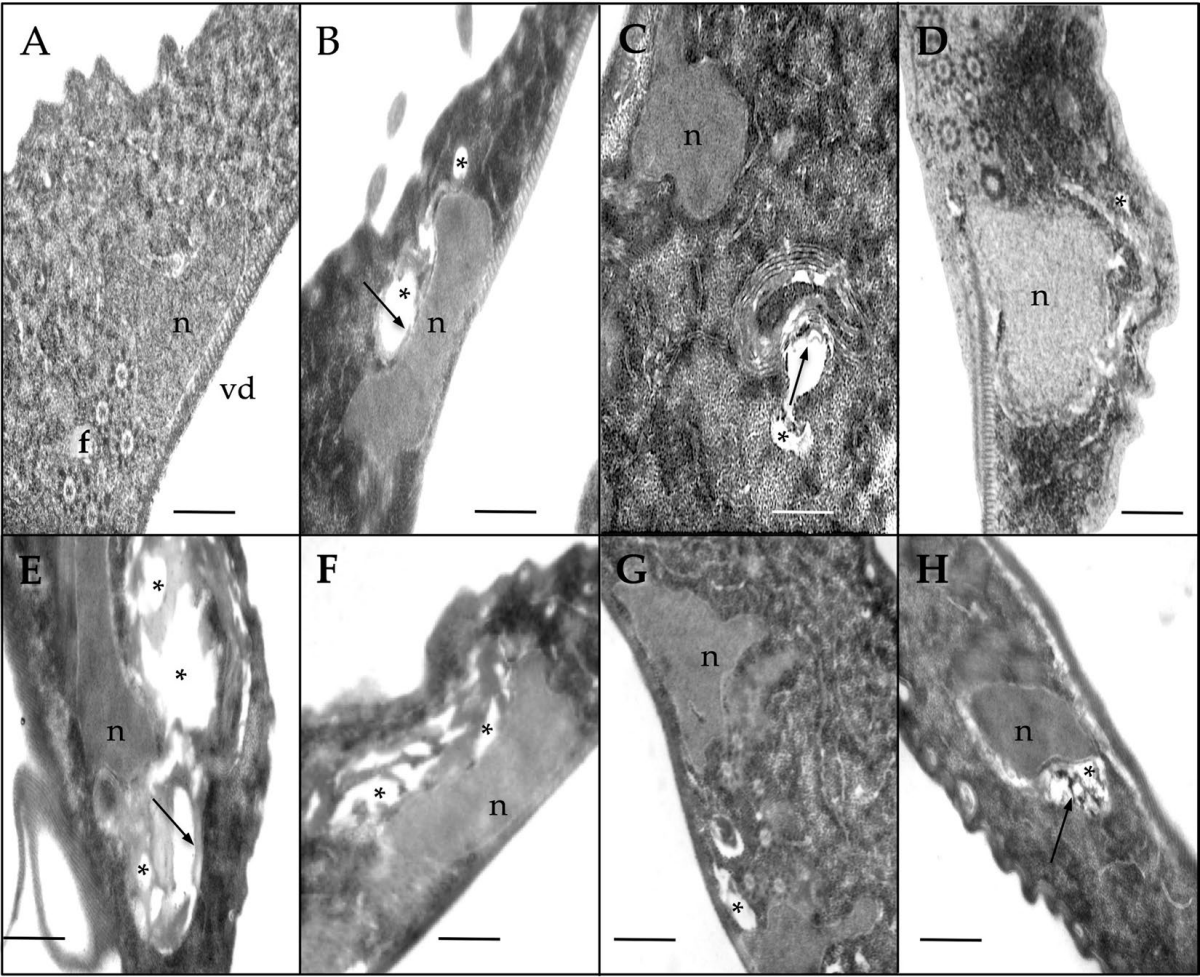
Ultrastructural effects of omeprazole (**B**–**D**), metronidazole (**E,F**), and nitazoxanide (**G,H**) on *Giardia* trophozoites. Control trophozoites were incubated with 0.05% DMSO (**A**). The ventral disk (vd), one of the two nuclei (n), and flagella (transversally cut) (f) are visible. Cells treated with omeprazole at 0.072 (**B**), 0.144 (**C**), and 1 μM (**D**). Cells treated with metronidazole at 1.24 (**E**) and 2.48 μM (**F**). Cells treated with nitazoxanide at 0.016 (**G**) and 0.032 μM (**H**). Asterisks show drug-induced vesicles. Arrows show lamellar structures. All treatments lasted 24 h independent of the compound used. Bar, 0.5 μm.

Previously, we reported the IC50 values for *G. lamblia* trophozoites with OMP, metronidazole (MTZ), and nitazoxanide (NTZ)^21^. Based on those results, we tested the lowest IC50 value previously calculated by us^21^ and the two-fold concentration for each drug (OMP, MTZ, and NTZ at 72 and 144 nM, 1.24 and 2.48 μM, and 16 and 3 nM, respectively) on *G. lamblia* trophozoites exposed for 24 h. An additional treatment of 1 μM OMP was included because this concentration can be reached in the plasma of patients receiving OMP treatment at a dosage of 20 mg/day^28,29^. Treated trophozoites exhibited important morphological changes compared with the control (Fig. 5A). OMP treatment induced cytoplasmic vacuolization even at the lowest concentration (72 nM) (Fig. 5B), and lamellar structures appeared at higher OMP concentrations (144 nM) (Fig. 5C). The general morphologic damage exerted by OMP at concentrations that can be reached in plasma is shown in Fig. 5D. In addition to the previously reported structural effects in *Giardia* trophozoites treated with MTZ^30^ and NTZ^31^, we observed similarities in cytoplasmic vacuolization between parasites treated with OMP and those treated with MTZ and NTZ (Fig. 5E–H). Apparently, the ventral disk and flagella did not appear to be damaged by OMP.

### High doses of omeprazole induce severe structural damage and cell death in *Giardia* trophozoites

**Figure 6:**
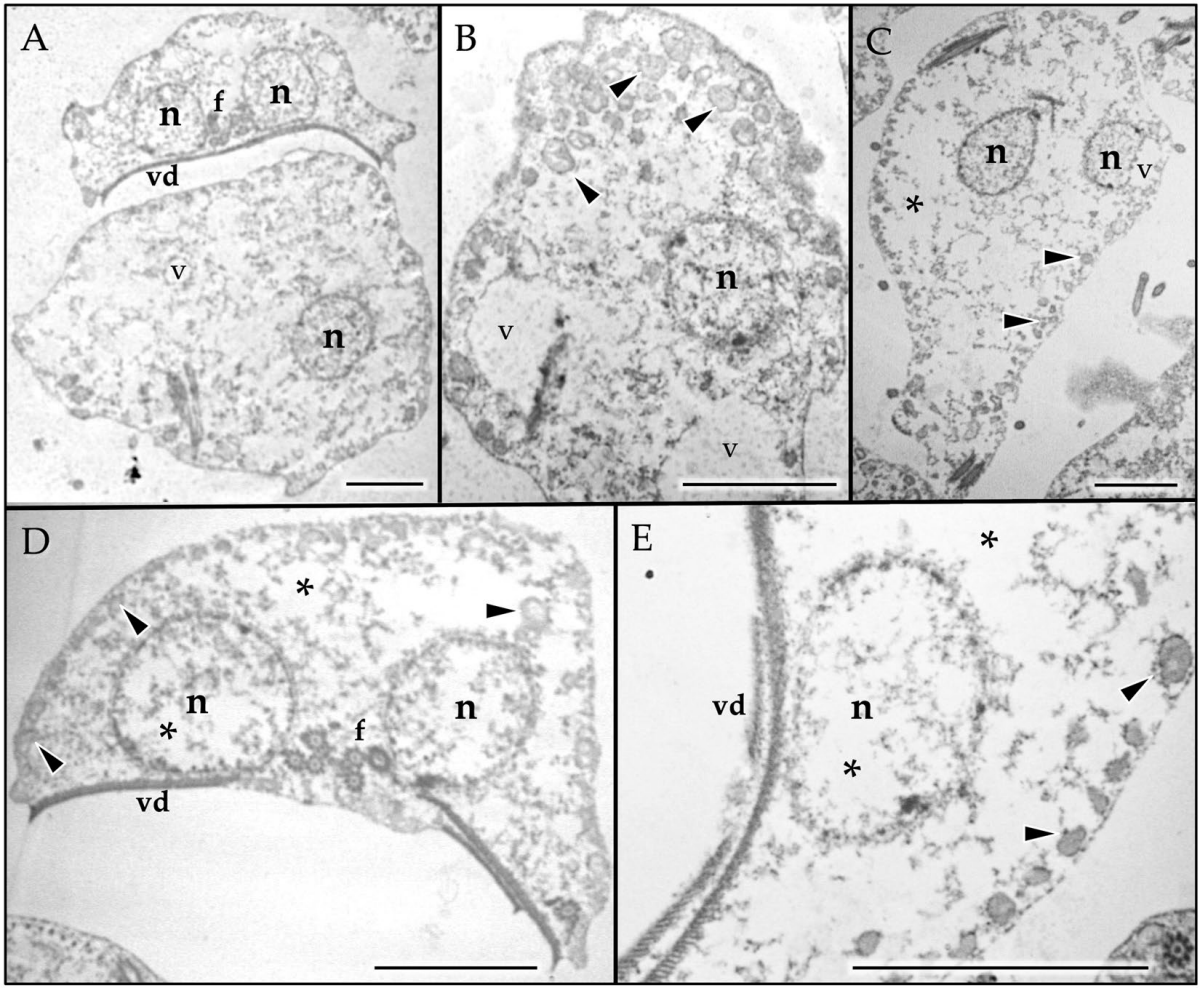
Ultrastructural effects of prolonged high doses of omeprazole on *Giardia* trophozoites. Trophozoites incubated for 24 h with 250 μM (**A–C**) and 500 μM OMP (**D,E**). Rounded giant trophozoites (**A**), intense vacuolization with at least two different types of vacuoles (**A**–**E**, arrowheads and v), and cytoplasmic and nuclear emptying (**A–E**, asterisks) were the observed effects of OMP. Nucleus (n), ventral disk (vd), flagella (f). Bar, 2.5 μm.

It has been proposed that the presence of lamellar structures, such as those observed in the treatments with OMP (Fig. 5B–D), suggests the induction of autophagy^32^. Deregulation of autophagy can initiate cell death under prolonged stress conditions similar to those exerted by OMP with prolonged treatment and when the overall concentration increases, which might occur when using OMP as a treatment against giardiasis. OMP treatment for 24 h at concentrations of 250–500 μM induced drastic morphological damage with obvious cytoplasmic and nuclear emptying (Fig. 6). The appearance of rounded giant trophozoites was observed even with 250 μM OMP treatment (Fig. 6A), accompanied by intense vacuolization with at least two different types of vacuoles (Fig. 6B,C). One of these vacuole types resembled peripheral vacuoles (PVs), which are related to vesicular trafficking in trophozoites (Fig. 6B–D, arrowheads), with electron-dense material in their inner lumen and electron-dense precipitates surrounding them. These electron-dense precipitates seem to be similar to those described when *Giardia* is treated with bismuth subcitrate^33^. The second vacuole type was larger, with a less electron-dense inner region than the previous type (Fig. 6, v). These vacuoles were frequently observed along with nuclear membrane blebbing (Fig. 6C, v), resembling the effect of oxidative stress induction^34^. Treatment with 500 μM OMP drastically increased the cytoplasm and nuclear emptying (Fig. 6D,E, asterisks) but conserved the vacuoles that appeared similar to PVs (PV-like) (Fig. 6D,E, arrowheads).

### Omeprazole also might target structural proteins

**Figure 7:**
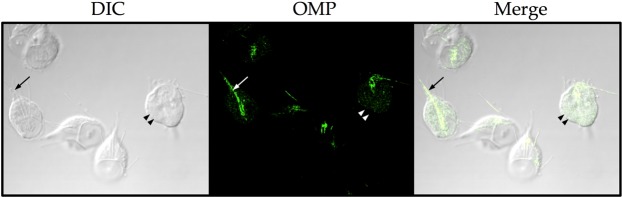
Confocal microscopy of *Giardia* trophozoites incubated with 100 μM omeprazole for 6 h. Fluorescence is attributed to omeprazole-protein adducts (panel OMP), which seem to localize in the cytoplasm as soluble proteins (double arrowheads) and in the flagella (arrow). Magnification, ×60. DIC, differential interference contrast.

We previously proposed the possibility of *in situ* localization of OMP-protein adducts due to the fluorescent property of OMP obtained when it establishes disulfide linkages with *Gl*TIM^21^. Figure 7 shows internalization of OMP into *Giardia* trophozoites. Actually, this property is shared by other compounds proposed to have antigiardial potential^35^. Interestingly, the OMP *in situ* distribution suggests not only interaction with soluble proteins (double arrow heads) but also structural proteins, such as those in flagella (Fig. 7, arrow). We did not observe damage to the microtubular structures of the trophozoites (*i.e*., the flagella and ventral disk), but this does not rule out the possibility of interaction between OMP and those structures.

**Figure 8:**
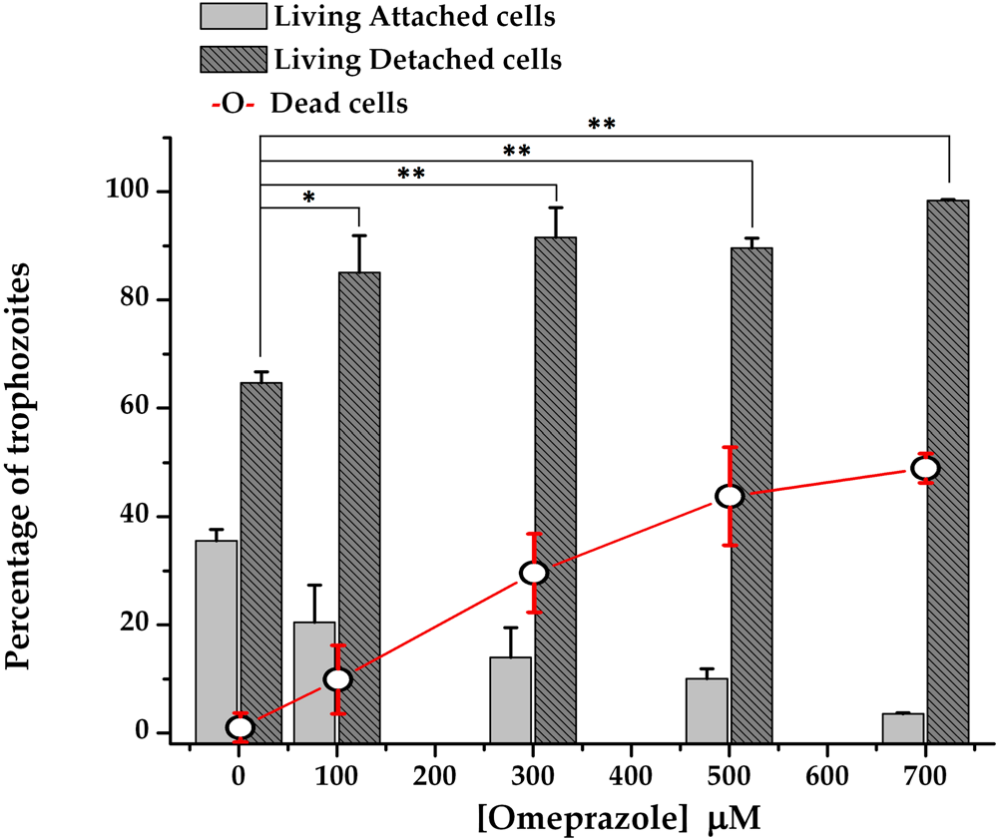
Effect of omeprazole on adherence and viability of *Giardia* trophozoites. Cells were incubated in the presence of increasing concentrations of OMP for 24 h, and then, trypan blue staining was used to distinguish between living and dead cells. Bars show the percentages of attached and detached cells according to the total number of living cells, whereas symbol + line shows the percentage of dead cells according to the total number of cells under each condition. The results are presented as the mean ± standard error of three independent experiments. Differences in the number of living detached cells between the control and treatment groups represent the effect on adherence. ANOVA and a Tukey–Kramer test showed significant differences between control and all treatment concentrations; *p < 0.05, **p = 0.001.

It is clear that the flagella and ventral disk are essential for parasite motility and attachment to host intestinal epithelial cells. Thus, based on the observed interaction of OMP with those structures, we analyzed the possibility that OMP could exert a functional affectation. To accomplish this, we analyzed the effect of OMP on the adhesion capacity of trophozoites. Figure 8 shows the dose-dependent deleterious effect of OMP on the adherence capacity of *Giardia* trophozoites. These results reinforce our hypothesis that OMP might also target structural proteins in the cytoskeleton.

## Discussion

With the growing concern about antibiotic resistance, there has been a strong push to develop novel experimental strategies and evaluate new drugs or repurpose known drugs^36,37^. However, the introduction of new drugs has been followed by evolution leading to resistance, and resistant strains can emerge within only a few years after the introduction of a drug to the clinic^38^.

Effective approved antigiardial drugs consist of six classes of compounds, namely, 5-nitroimidazole and benzimidazole derivatives, quinacrine, furazolidone, paromomycin, and nitazoxanide, but *G. lamblia* has developed resistance to all of them, even developing multidrug resistant strains^39–41^. Among the drug resistance mechanisms present in *G. lamblia*, acquisition of nonsynonymous mutations in several genes from resistant lines has been demonstrated^42^, which can yield amino acid substitutions and avoid the deleterious effect of antigiardial drugs^43^.

From the so-called new promising anti-*Giardia* drugs^37^, OMP is one of the most safe and low-cost drug that has been efficiently repurposed for use against *G. lamblia*. In a previous work, we showed evidence supporting the proposed concomitant *Gl*TIM inhibition and cytotoxic activity exerted by OMP in *Giardia*^21^. The inhibition exerted by OMP on G*l*TIM is primarily based on its binding to the cysteine residue at position 222^21,23^. To determine whether the inhibition of G*l*TIM previously described is important in the effect of OMP on *Giardia*, while considering the possibility that the parasite could mutate TIM to avoid the antigiardial effect of this proton pump inhibitor, we examined the *in vitro* potential of OMP to inhibit *Gl*TIM mutants lacking the most accessible Cys.

To better understand the overall results, it is important to note that the reported concentrations used in this study to inhibit recombinant G*l*TIM are much higher than the IC50 values calculated in trophozoites (116 nM)^21^. This is due to the conditions needed to achieve an equimolar reaction in both cases; thus, 2 h time-lapse assays of purified, highly concentrated recombinant G*l*TIM require high concentrations of OMP, whereas 24-h time-lapse assays of cellular G*l*TIM require much lower concentrations of OMP.

Since the catalytic efficiency of the produced recombinant mutants was in the same range, we claim that substitutions of Cys at positions 202, 222 and 228 by alanine (Ala) (Table 1) do not affect the catalytic center of *Gl*TIM and that the lack of these Cys is not an impediment to generation of functional enzymes. Even the variability in Tm between WT and the mutants is in the range of the natural variability of mesophilic proteins^44^. Nonetheless, it is remarkable that some features of these enzymes could lead disadvantages for the parasite. On a related note, the kinetics of Tmut were affected, resembling those of the *Gl*TIM tetramer, with a *k*_cat_ of approximately half that of the dimer^22^; this finding might be because the 3 superficial Cys residues studied here are related to oligomerization and enzyme activity modulation^45^. Similarly, the catalytic efficiency (*k*_cat_/*K*_m_) of Tmut also resembled that of the *Gl*TIM tetramer; moreover, the overall kinetics of Tmut were similar to those of *Gl*TIM derivatized with the thiol reactive compound MMTS^19^. On the other hand, Dmut showed a remarkably increased affinity for the substrate, *i.e*., double that of the WT enzyme. This could be advantageous for the parasite, but if these two Cys (222 and 228) are needed to regulate the enzyme activity of *Gl*TIM in the cyst, such a mutation in *Gl*TIM would affect the normal encystation process.

We previously proposed that *Gl*TIM oligomerization is of functional significance at some stages of the life cycle of *G. lamblia*^45^; therefore, effects on this oligomerization process may confer disadvantages to strains of *Giardia* that mutate as a result of selective pressure. From the results revealed here, we can conclude that production of a functional *Gl*TIM without the most superficial Cys is feasible but could represent a disadvantage to parasites carrying such mutations. Moreover, these mutations would be insufficient to avoid the inhibitory effect of OMP on *Gl*TIM.

Our results show that OMP can inhibit *Gl*TIM (WT and mutants) even when this enzyme is in a heterologous cell, such as a bacterium. The cytotoxicity observed in bacteria transformed with *Gl*TIM suggests that, rather than an energetic imbalance, this effect provoked by OMP might be mediated through intoxication due to high production of methylglyoxal, a nonenzymatic product of DHA, which exerts irreversible effects on protein structure and function.

Based on the above findings, if binding of OMP with *Gl*TIM contributes to the cytotoxicity exerted against *Giardia*, then a plausible explanation might be based on the following: (1) the conformational stability of *Gl*TIM is strongly affected by OMP^23^; (2) the factors that naturally affect the stability of other TIMs lead to accumulation of methylglyoxal^46^; and (3) *G. lamblia* does not have two important enzymes in the main catabolic route for methylglyoxal: glyoxalase 1 and 2^47,48^. Methylglyoxal is recognized as a toxic metabolite that can induce apoptosis^49^, due to malfunction of triosephosphate isomerase^50^. Moreover, methylglyoxal is a potent precursor of advanced glycation end products^46^. Therefore, we hypothesize that the increased formation of AGEs as a result of methylglyoxal accumulation might (at least in part) be related to the cytotoxic effects of OMP.

Regarding the possibility of OMP targeting glycolysis in *Giardia*, we consider the following. If the use of glucose for energy generation were affected, then glucose levels would be expected to rise in trophozoites. Excess glucose is stored as glycogen in *G. lamblia*^51^, and glycogen accumulates when glucose levels are elevated^52^. When we compared the glycogen staining patterns of wild-type trophozoites with those of trophozoites treated with OMP, the results showed an increase in glycogen deposition and further support the suggestion that OMP affects energy generation through glucose utilization. Since salivary amylase hydrolyzes glycogen, the disappearance of PAS-positive granules from trophozoites (applied after OMP treatment and before PAS staining) also supports this idea.

This is the first work in which the effects of OMP on *Giardia* trophozoite morphology were analyzed. The effectiveness of this PPI was comparable at the ultrastructural level to those of MTZ and NTZ, and the effects were observed at low concentrations where lamellar structures related to autophagy were observed. The most drastic morphologic damages were observed at the highest range of OMP concentrations tested. We hypothesize that this effect could be reached during conventional treatment with 38 mg/day of OMP, but further studies in animal models must be done to confirm this dosage. Additionally, to demonstrate the morphologic damage exerted by OMP, we also show its effect on the adherence of trophozoites in culture. Taking into account the overall results, including analysis of the fluorescence of OMP-treated trophozoites, we consider that OMP may target *Gl*TIM and consequently affect glycolysis, but it is possible that the cytotoxicity that OMP exerts toward *Giardia* trophozoites may be the sum of the impairment of several molecules and/or metabolic processes that together are important for the survival of this parasite. Based on the observed effects on viability and adherence, we postulate that *Giardia* adherence is also affected by omeprazole; however, cell death appears to occur faster than detachment, which did not permit detection of a linear dose-dependent effect.

It is important to note that our proposal hypothesizes that G*l*TIM is one target of omeprazole in *Giardia* and that its alteration could potentially (sooner or later) contribute to the cytotoxicity exerted by this drug. Certainly, we cannot assure that OMP activity against *Giardia* depends on its binding affinity to G*l*TIM because other molecules might be critical factors in the cell death process observed in this study. However, we also cannot completely discard the possibility that the effect of OMP on G*l*TIM contributes to its cytotoxic effect. On the other hand, these findings reinforce the use of OMP as an alternative drug for giardiasis treatment, but the search for the molecules it targets and their specific contribution on the effect of OMP against *Giardia* must be further studied to develop more efficient and selective treatments.

## Materials and Methods

### Reagents and general materials

Unless otherwise specified, all reagents were purchased from Sigma-Aldrich (St. Louis, MO, USA), including omeprazole. Luria-Bertani (LB) medium and isopropyl-β-D-thiogalactopyranoside (IPTG) were purchased from AMRESCO LLC (Cochran Road Solon, OH, USA). Glycerol-3-phosphate dehydrogenase (α-GDH) and reduced nicotinamide adenine dinucleotide (NADH) were purchased from Roche (Penzberg, Upper Bavaria, Germany). T7 oligonucleotides were from Novagen, and Phusion polymerase was from Thermo Scientific. Immobilized metal affinity chromatography (IMAC) resin was purchased from Bio-Rad (Hercules, California, USA). Amicon Ultra 30 kDa filters were purchased from Millipore Corporation (Billerica, Massachusetts, USA).

### Construction of mutants

We generated double and triple Cys mutants via site directed mutagenesis^53^ using the previously constructed *C228A gltim* mutant gene as a template^45^. For all the mutants, T7 promoter and T7 terminator oligonucleotides were used. Mutagenic primers to obtain the double and triple mutated genes were as follows:

C222-Fwd: 5′-GGAAGCAACGCTGAGAAGC-3′-Rev: 5′ GCTTCTCAGCGTTGCTTCC-3′;

C202-Fwd: 5′-GGAGAAGGTTGCTGCCG-3′-Rev: 5′-CGGCAGCAACCTTCTCC-3′.

Using the C228A *gltim* mutant gene (contained in pET-3aHisTev plasmid) as template and mutagenic primers for C202, the double mutant *C2*0*2/228A gltim* gene was constructed. Using the *C202/228A gltim* mutant gene (contained in pET-3aHisTev plasmid) as template and mutagenic primers for C222, we constructed the triple mutant *C202/222/228A gltim* gene. PCR products were purified by electrophoresis using an extraction kit (QIAquick, QIAGEN) and separately cloned into a pET-3aHisTev plasmid by using the Nde1 and BamH1 restriction sites and were ligated by incubation with T4 DNA ligase (New England Biolabs) at 16 °C overnight.

The PCR mixtures contained 100 ng of the *C228A or C202/228A gltim* genes, 1.5 mM MgCl_2_, 0.2 mM dNTPs, and 2.5 U of Phusion polymerase. Amplification was performed at 98 °C for 4 min, followed by 25 cycles at 98 °C for 1 min, 55 °C for 1 min, and 72 °C for 1 min, with an added final extension at 72 °C for 10 min. The genes encoding *Gl*TIM mutants were completely sequenced to verify the desired sequence (data not shown). Competent *E. coli* cells, strain BL21(DE3)pLysS, were transformed with pET-3aHisTev- *C202/228A* or pET-3aHisTev- *C202/222/228A* by using heat shock.

### Purification of *Gl*TIM WT and *Gl*TIM double and triple Cys mutants

Bacteria containing the plasmids for *Gl*TIM WT or the *Gl*TIM Cys mutants were grown separately in LB medium supplemented with 0.1 mg/mL ampicillin and incubated at 37 °C. When the cultures reached an Abs_600_ = 0.8, they were induced using 0.4 mM IPTG and incubated overnight at 30 °C with shaking at 180 rpm. After induction and growth, the bacteria were collected by centrifugation (6500 rpm, 15 min) and suspended in 40 mL of lysis buffer (pH 8.0) containing 50 mM Tris, 50 mM NaCl, 5 mM β-mercaptoethanol, and 1 mM phenylmethylsulfonyl fluoride (PMSF). The bacterial suspension was disrupted by sonication and centrifuged at 9000 rpm for 1 h at 4 °C. Protein purification was performed via IMAC using a Profinity Ni^2+^ charged resin previously equilibrated with lysis buffer. The soluble protein fraction was mixed with the equilibrated Ni^2+^ charged resin and incubated at room temperature with shaking for 30 min. Then, the column was washed with the same buffer (10 column volumes) to remove proteins without the His-tag sequence. The desired proteins were eluted with lysis buffer containing 200 mM imidazole, adjusted to pH 8.0. The purified proteins were concentrated using Amicon ultrafiltration units. The purity of the enzymes was analyzed via sodium dodecyl sulfate-polyacrylamide gel electrophoresis (16% SDS–PAGE gel) and stained with colloidal Coomassie Brilliant Blue. The enzyme concentration was spectrophotometrically determined (Spectrophotometer Cary 50, Varian Inc) at 280 nm using the extinction coefficient ε_280_ = 26,600 M^−1^ cm^−1^.

### Kinetics

The enzymatic activities of the purified recombinant *Gl*TIM WT and *Gl*TIM Cys mutants were determined by monitoring the conversion of glyceraldehyde 3-phosphate (GAP) to dihydroxyacetone phosphate (DHAP). Briefly, the conversion of GAP to DHAP using α-GDH as the coupling enzyme was followed spectrophotometrically by monitoring NADH oxidation at 340 nm (Spectrophotometer Cary 50, Varian Inc). Determination of *K*_m_ and *V*_max_ was achieved by fitting initial velocity data at GAP concentrations ranging from 0.3 to 3 mM to the Michaelis-Menten equation (*V*_*0*_ = *V*_max_ • S/*K*_m_ + S) by using nonlinear regression calculations. The *k*_cat_ was derived from *V*_max_ by considering a molecular mass for the monomer of 27.7 kDa.

### Circular dichroism and thermal unfolding experiments

CD measurements at the far UV region (190–240 nm) of *Gl*TIM WT, Dmut, and Tmut were performed on 0.2 mg/mL samples with a J-810 spectropolarimeter (Jasco) equipped with a Peltier thermostatted cell holder in a 0.1-cm quartz cell at 25 °C. The background buffer spectrum was obtained under the same experimental conditions (without protein) and was always subtracted from the data.

Thermal unfolding studies were performed using 0.2 mg/mL protein samples to monitor the protein ellipticity at 222 nm with the same spectropolarimeter increase from 40 to 70 °C at a rate of 1 °C/2.5 min in TED buffer pH 7.4 (100 mM triethanolamine, 10 mM EDTA). The denatured fraction (*f*D) was calculated using the equation *f*D = (*y*N − *y*) ⁄ (*y*N − *y*D), where *y*N and *y*D are the ellipticity values for the native and unfolded proteins, respectively. These parameters were determined from the initial and final data of the *y versus* temperature curve, and the unfolding temperature (Tm) value was obtained from the aforementioned equation.

### *In vitro* inhibition of *Gl*TIM WT and *Gl*TIM Cys mutants

To explore the degree of *Gl*TIM inhibition by OMP *in vitro*, assays were performed at an enzyme concentration of 0.2 mg/mL (7.2 nM) in triethanolamine-EDTA (TE) buffer with the indicated concentrations of omeprazole during 2 h of incubation at 37 °C. Aliquots from these incubations were withdrawn and assayed for residual activity with the standard reaction mixture. The standard reaction mixture contained 1 mM GAP, 0.2 mM NADH, and 0.9 units/mL of α-GDH in TE buffer. The reaction was initiated by adding 5 ng/mL TIM to the reaction mixture. The data are reported as the percentage of residual activity, using the activity of each enzyme incubated without OMP as 100% activity. All compounds and enzymes were prepared and diluted immediately before use. It is important to note that the high concentrations of OMP used in these assays were necessary to obtain conditions close to equimolarity between OMP and recombinant G*l*TIM. Thus, note that recombinant G*l*TIM was at a high concentration, whereas cellular G*l*TIM was at a much lower concentration. Additionally, high concentrations of OMP are needed when the incubation times are short.

### *In situ* inactivation of *Gl*TIM WT and *Gl*TIM Cys mutants and effect on bacterial viability

Bacteria cultures transformed with either wt, double, or triple mutant *gltim* were cultured separately until reaching an Abs_600_ = 0.8. Cultures were induced with IPTG and incubated overnight as described above. After induction and growth, the cultures were incubated for 24 h with the indicated concentrations of OMP. The recombinant enzymes were purified from bacteria cultures, and their enzyme activity was measured as described above.

To determine the effect of OMP on the viability of bacteria overexpressing *Gl*TIM WT or the *Gl*TIM Cys mutants, bacteria cultures were prepared as above and aliquots from each culture were used at 1:2000 dilution with freshly sterilized LB medium, and 0.1 mL was plated onto plate count agar with 100 μg/mL ampicillin. Bacteria transformed with pET-3aHisTev without an incorporated *gltim* gene were used as the control. Plates were incubated for 15 h at 37 °C, colonies were counted, and CFU/mL were calculated using the following formula: CFU/mL = (no. of colonies × dilution factor)/volume of culture plate. The results are reported as the percentage of surviving bacteria, considering bacteria from each culture without OMP as 100%.

### *Giardia* trophozoites

Trophozoites of *G. lamblia* WB strain were cultured in TYI-S-33 as previously described^22^. Cultures were grown at 37 °C for 72 h until reaching log phase growth and then incubated with the indicated concentrations of OMP. Cultures contained between 14 × 10^6^ and 16 × 10^6^ trophozoites (~2 × 10^6^–2.3 × 10^6^ trophozoites/ml of culture medium) at the moment when the incubation with omeprazole was initiated.

### Glycogen staining

*Giardia* trophozoites were grown to confluence on coverslips in six-well cell culture plates (Costar) with TYI-S-33 medium for 48 h. After this, the coverslips with a monolayer of trophozoites were incubated with 100 μM OMP for 6 h or with 500 μM OMP for 1 h. Trophozoites on coverslips without OMP were also incubated for 6 or 1 h as controls. After incubation with OMP, the cells were washed three times (10 min each) with phosphate-buffered saline (PBS; pH 7.2) at room temperature and fixed overnight at 4 °C in 2% paraformaldehyde (w/v) in 50 mM cacodylate buffer (pH 7.2). After fixation, the coverslips were washed 3 times with PBS (10 min each) at room temperature, permeabilized in 0.2% Triton X-100 in PBS for 5 min at 4 °C, and again washed 3 times with PBS. Some of the coverslips treated with OMP were incubated with amyloglucosidase (2 mg/mL, *i.e*., 51 U/mg) prior to PAS staining. Glycogen staining was performed as previously described^54^. Briefly, samples were oxidized with 0.5% periodic acid for 5 min, rinsed in distilled water, incubated with Schiff reagent for 15 min, washed in lukewarm tap water for 5 min, counterstained with Mayer’s hematoxylin for 1 minute, dehydrated and mounted.

### Ultrastructural analysis

Trophozoites from each treatment were chilled on ice for 20 min and centrifuged to obtain a cell pellet containing more than 10 × 10^6^ trophozoites each. The pellets were fixed with 2.5% glutaraldehyde in 0.1 M PBS (pH 7.2) at 4 °C for 24–48 h and post fixed with 2% osmium tetroxide in the same buffer for 60 min. After dehydration with increasing concentrations of ethanol and propylene oxide, samples were embedded in epoxy resin and polymerized at 60 °C for 24 h. Ultrathin sections of 60 nm were counterstained with uranyl acetate and lead citrate. Micrographs were obtained with a JEOL-1011 transmission electron microscope.

### Confocal microscopy

Trophozoites were grown on glass coverslips in six-well cell culture plates (Costar) with TYI-S-33 medium for 48 h followed by incubation for 6 h with 100 μM omeprazole diluted in TYI-S-33 medium. Afterward, the coverslips were washed three times with PBS (5 min each) and mounted with mounting medium for fluorescence analysis (Vectashield; Vector). The mounted samples were irradiated for 15 min in a UV chamber (homemade with a fluorescent black-light tube; WKO model F8T5BL) and immediately analyzed and photographed with a laser scanning confocal microscope (Olympus FV1000) using a 405-nm violet diode laser.

### Effect of OMP on the adhesion capacity of *Giardia* trophozoites

After treatment with 100, 300, 500, and 700 μM OMP for 24 h, culture tubes were decanted to separate attached cells from detached cells. Culture tubes were refilled with cold PBS and chilled on ice for 20 min. The number of detached and attached cells was determined microscopically using a hemocytometer (Neubauer cell-counting chamber). Additionally, dead cells were quantified using trypan blue staining. The results are expressed as the percentage of attached and detached trophozoites in relation to the total number of living cells per culture tube. The number of dead trophozoites is expressed as a percentage relative to the total cell number per culture tube (dead and living cells).

### Measurement of free methylglyoxal

Methylglyoxal (MGO) levels were determined by using 2,4-dinitrophenylhydrazine (DNPH) according to Gilbert and Brandt^55^ with modifications.

Briefly, 2 × 10^6^ *G. lamblia* cells untreated or treated with OMP (580 µM) for 24 h at 37 °C were cooled, washed three times with ice-cold PBS, and lysed via five freeze/thaw cycles (liquid nitrogen and 45 °C, respectively). Next, was added ice-cold 2 M perchloric acid (PCA), and the samples were incubated on ice for 10 min and centrifuged (12,000 rpm, 4 °C, 10 min). The supernatant was removed and stored at 4 °C for further measurement.

Before determining the concentration of MGO in the samples, standard values of MGO were calculated by using the DNPH method. Stock solutions of 20 mM DNPH in HCl-ethanol (12:88) and 1 mM MGO (Sigma) in water were prepared. Increasing concentrations of MGO (0 to 25 µM) were incubated with 0.2 mM DNPH at 42 °C for 45 min, the samples were cooled for 5 min at room temperature, and the absorbance of bis-2,4-dinitrophenyl-hydrazone of MGO was recorded at 432 nm with a Cary 50 UV/VIS spectrophotometer (Agilent technologies, California USA). Finally, MGO from *G. lamblia* supernatants were assayed using the mentioned method. The MGO concentrations were calculated according to an extinction coefficient of ε = 33,600 M^−1^ cm^−1^ for bis-2,4-dinitrophenyl-hydrazone.

### Quantification of advanced glycation end products (AGEs)

AGE levels were measured using an advanced glycation end products (AGEs) ELISA Kit from MyBioSource, San Diego, CA (United States). This experiment employed the double-sandwich ELISA technique, and the ELISA Kit utilized is typical. The precoated antibody was an AGE monoclonal antibody, and the detection antibody was a biotin-labeled polyclonal antibody. Samples and the biotin-labeled antibody were added into the ELISA plate wells, and unbound molecules were washed with PBS. Then, avidin-peroxidase conjugates were added to the ELISA wells in order. The 3,3′,5,5′-tetramethylbenzidine (TMB) substrate for coloring was added after reactant was thoroughly washed with PBS. TMB turns blue in the presence of peroxidase catalytic activity and finally turns yellow under the action of acid. The color depth and the testing factors in the samples are positively correlated.

A standard curve was constructed with standard AGE samples at the following concentrations: 200, 100, 50, 25, 12.5, 6.25, and 3.12 ng/ml. Samples were obtained from *Giardia* lysates, taken at a concentration of 1 µg/µL, subsequently diluted at a ratio of 1: 100 and loaded into the ELISA plate for determination of the AGE concentration.

### Statistical analysis

All results are expressed as the means ± standard deviations (SD). All data were analyzed using the NCSS 2007 (Number Cruncher Statistical System) statistical software package program (Utah, USA). Statistical comparisons were performed using one-way analysis of variance (ANOVA) followed by Tukey’s test. *p* values < 0.05 were considered statistically significant.

## Supplementary information

Supplementary Figures

Editorial Certificate of Languaje
