# Supplementary material for: On the molecular and cellular effects of omeprazole to further support its effectiveness as an antigiardial drug
Source: Sci Rep. 2019 Jun 20;9:8922. doi: 10.1038/s41598-019-45529-w (PMC6586891; doi:10.1038/s41598-019-45529-w)
Supplement: Supplementary file 1 — Supplementary Figures [file 41598_2019_45529_MOESM1_ESM.pdf]

On the molecular and cellular effects of omeprazole to further support its effectiveness as an antigiardial drug.

Gabriel López-Velázquez, Cynthia Fernández-Lainez, José Ignacio de la Mora-de la Mora, Daniela Caudillo de la Portilla, Rafael Reynoso-Robles, Angélica González-Maciel, Cecilia Ridaura, Itzhel García-Torres, Pedro Gutiérrez-Castrellón, Alfonso Olivos-García, Luis Antonio Flores-López, Sergio Enríquez-Flores

Supplementary Figures.

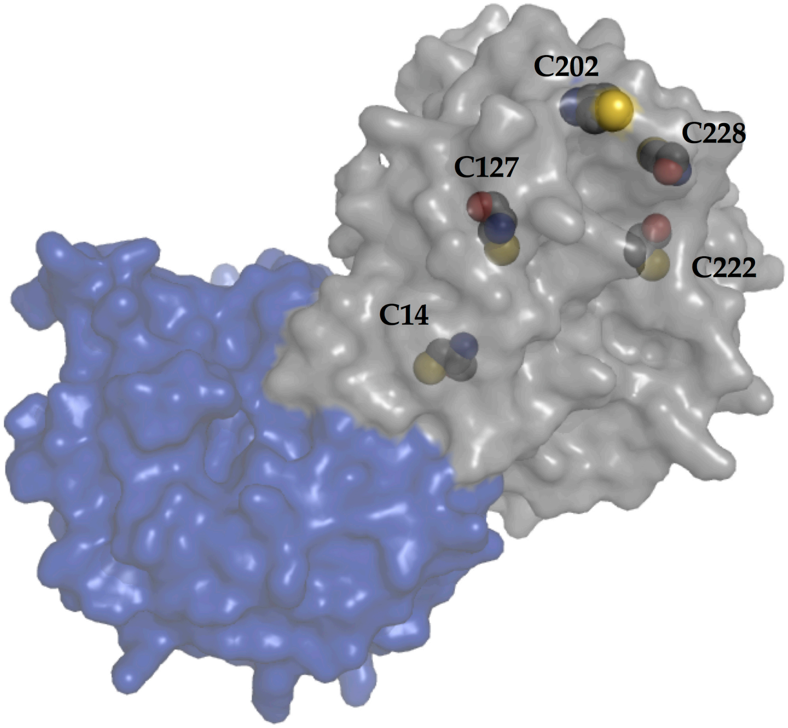

Figure S1. 3D structure of G*l*TIM dimer and the distribution of its five cysteines per monomer. Modeled with surface representation (PyMOL 2.3). Each dimer subunit is differentiated by color (gray and blue). C14 is buried in the dimer interface. (PDB: 2DP3).

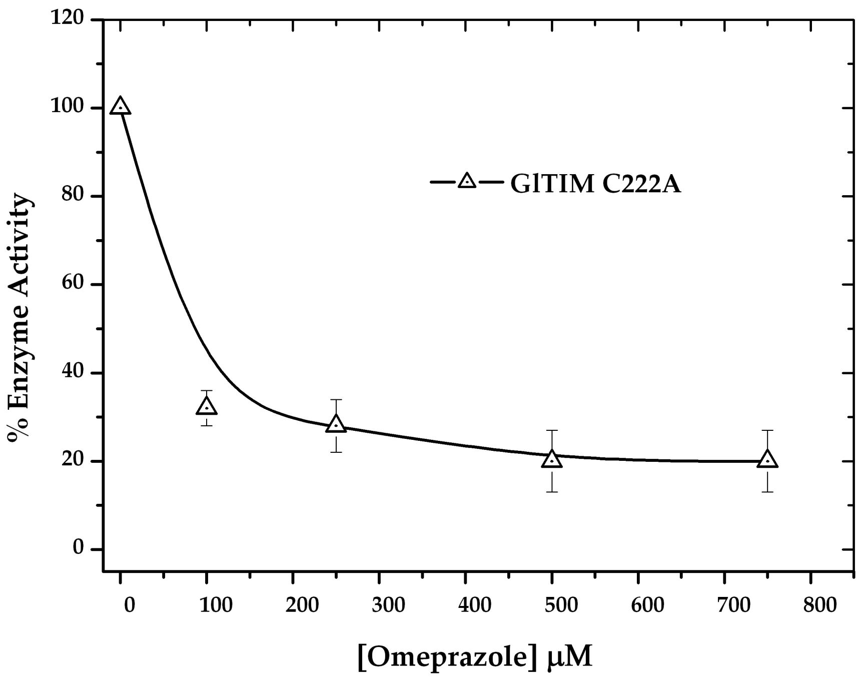

Figure S2. Effects of omeprazole on G*l*TIM C222A mutant after 24 h of incubation.

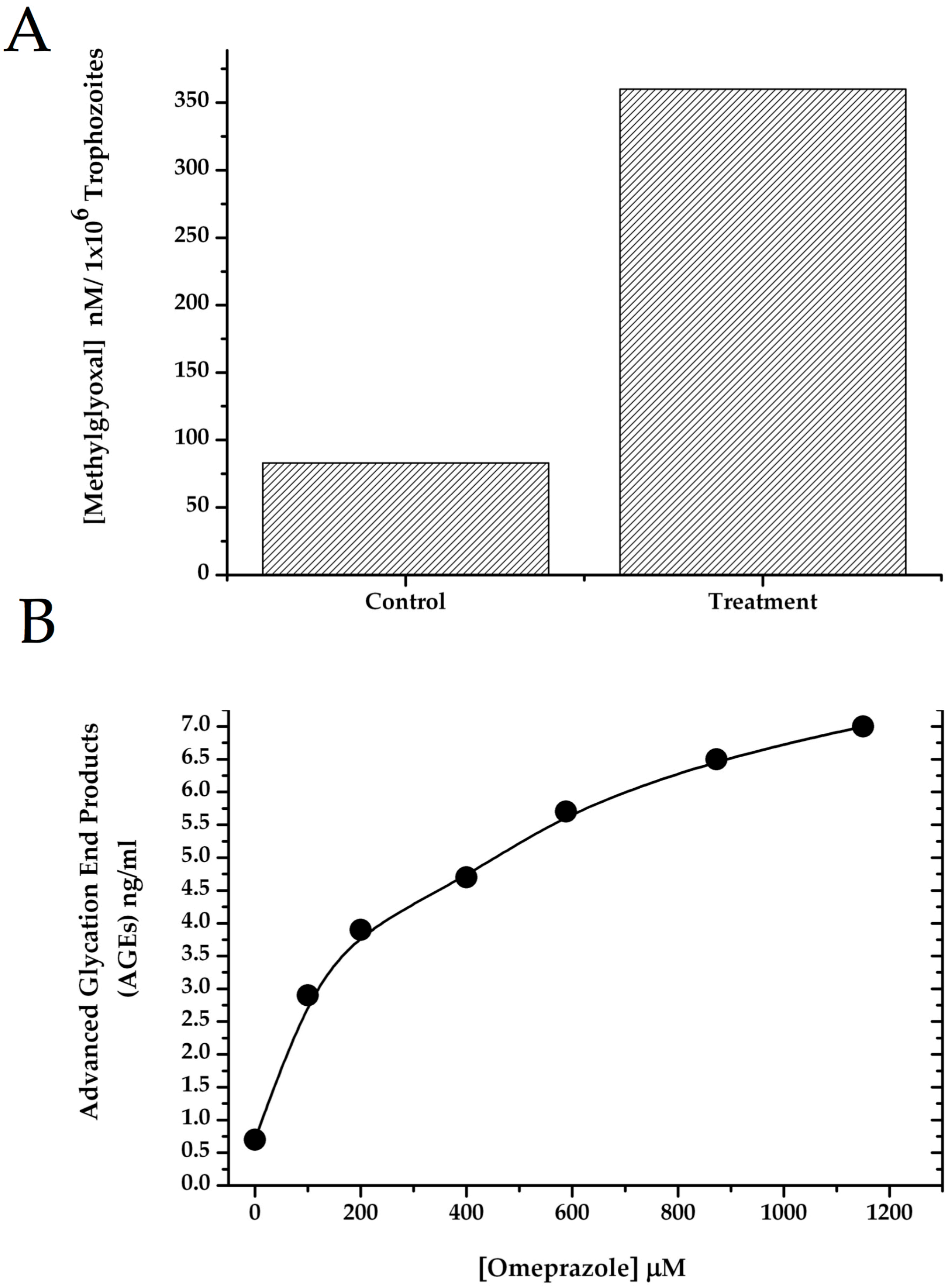

Figure S3. Increase of methylglyoxal (**A**) in trophozoites treated with 580 μM OMP with respect to not treated (control) and accumulation of AGEs in proteins of *Giardia* trophozoites treated with increasing concentrations of OMP (**B**).
